# The Relationship Between Lifespan Volunteerism and Personality Among Oldest Old Adults

**DOI:** 10.1093/geroni/igaa057.1265

**Published:** 2020-12-16

**Authors:** Gina Lee, Peter Martin

**Affiliations:** Iowa State University, Ames, Iowa, United States

## Abstract

There has been a lack of studies exploring volunteerism from the life span perspective. This study aims to examine the relationship between personality and volunteerism among the oldest old population, using three types of volunteerism: “ever volunteered,” “last volunteered,” and “currently volunteering.” “Ever volunteered” assesses whether individuals ever volunteered in their life. “Last volunteered” examines when an individual last volunteered. “Currently volunteering” explores whether an individual is currently volunteering. By comparing the three volunteerism measures, this study took a life span view of volunteerism. Data of 208 oldest old adults, octogenarians (34.1%) and centenarians (65.9%), from the Georgia Centenarian Study were included in this study. The majority of the sample had volunteered sometime during their lifetime (88.9%), many of them still volunteered when they were in their 80s and 90s (40.4%), and the majority of the sample indicated that they were not currently volunteering (78.8%). Multiple regression analyses indicated that competence (a facet of conscientiousness) significantly predicted “ever volunteered,” and extraversion significantly predicted “last volunteered.” In other words, oldest old adults with high competence levels were more likely to have volunteering experiences in their life. Also, those with high levels of extraversion were likely to have more recent volunteering experiences. None of five personality traits significantly predicted “currently volunteering.” This study sheds light on the importance of different types of volunteerism which enables us to better understand the relationship between volunteerism and personality. We recommend future research to test the link between different types of volunteerism and well-being outcomes.

